# Inferring Haplotypes of Copy Number Variations From High-Throughput Data With Uncertainty

**DOI:** 10.1534/g3.111.000174

**Published:** 2011-06-01

**Authors:** Mamoru Kato, Seungtai Yoon, Naoya Hosono, Anthony Leotta, Jonathan Sebat, Tatsuhiko Tsunoda, Michael Q. Zhang

**Affiliations:** * Cold Spring Harbor Laboratory, Cold Spring Harbor, NY 11724; † Center for Genomic Medicine, RIKEN, Yokohama, Kanagawa 230-0045, Japan; ‡ Department of Psychiatry, University of California, San Diego, La Jolla, CA 92093; § Bioinformatics Division, TNLIST/Department of Automation, Tsinghua University, Beijing, 100084, China

**Keywords:** copy number variation, EM algorithm, haplotype inference, phasing

## Abstract

Accurate information on haplotypes and diplotypes (haplotype pairs) is required for population-genetic analyses; however, microarrays do not provide data on a haplotype or diplotype at a copy number variation (CNV) locus; they only provide data on the total number of copies over a diplotype or an unphased sequence genotype (*e.g.*, AAB, unlike AB of single nucleotide polymorphism). Moreover, such copy numbers or genotypes are often incorrectly determined when microarray signal intensities derived from different copy numbers or genotypes are not clearly separated due to noise. Here we report an algorithm to infer CNV haplotypes and individuals’ diplotypes at multiple loci from noisy microarray data, utilizing the probability that a signal intensity may be derived from different underlying copy numbers or genotypes. Performing simulation studies based on known diplotypes and an error model obtained from real microarray data, we demonstrate that this probabilistic approach succeeds in accurate inference (error rate: 1–2%) from noisy data, whereas previous deterministic approaches failed (error rate: 12–18%). Applying this algorithm to real microarray data, we estimated haplotype frequencies and diplotypes in 1486 CNV regions for 100 individuals. Our algorithm will facilitate accurate population-genetic analyses and powerful disease association studies of CNVs.

Large-scale studies employing high-throughput experimental technologies have recently revealed the genome-wide nature of copy number variations (CNVs) (Conrad *et al.* 2010; Freeman *et al.* 2006; Iafrate *et al.* 2004; Kato *et al.* 2010; McCarroll *et al.* 2008; Redon *et al.* 2006; Sebat *et al.* 2004), which are variations of the number of DNA segments >1 kilobase in size, and it is estimated that CNVs occupy as much as 4–6% of the human genome. The importance of CNVs in phenotypic traits and disease susceptibility has been revealed in their associations with HIV infection, autoimmunity, autism, schizophrenia, and cancer (Merikangas *et al.* 2009; Sebat 2007; Speleman *et al.* 2008). Currently, high-throughput experimental technologies for CNVs cannot provide data on a haplotype or diplotype (haplotype pair) of CNVs; instead, they provide data on the total number of copies over a diplotype or an unphased sequence genotype (*e.g.*, AAB at a CNV locus, where A and B represent alleles) (Conrad and Hurles 2007; Kato *et al.* 2008a; Kato *et al.* 2008b). This unknown state of haplotypes hinders precise population-genetic analyses such as analyses of allele frequencies, linkage disequilibrium (LD), and population differentiation, as well as the development of efficient strategies for disease-association studies (McCarroll and Altshuler 2007).

To obtain information on haplotypes, algorithms and computational tools have been developed to infer haplotypes from total copy numbers or unphased sequence genotypes (Kato *et al.* 2008a; Kato *et al.* 2008b; Shindo *et al.* 2009; Su *et al.* 2010). In this regard, algorithms using similar procedures have also been developed to infer haplotypes from the total copy numbers or unphased genotypes of >2 DNA copies in polyploid chromosomes (Clark *et al.* 2004; Neigenfind *et al.* 2008; Su *et al.* 2008). These algorithms are based on the implicit assumption that total copy numbers or unphased genotypes are measured with good accuracy. While reasonable for data from target-specific assays such as quantitative PCR and the RETINA technique (Hosono *et al.* 2008), as was previously demonstrated (Hosono *et al.* 2009), it is not always true for data from high-throughput genome-wide platforms such as microarrays. Microarray data are noisy overall (McCarroll and Altshuler 2007) and replicate experiments to correct these inaccuracies often difficult to repeat. Noise in microarrays causes uncertainty in the determination of total copy numbers or unphased genotypes. For example, when greater noise is present in a signal intensity deriving from a total copy number of 1, the observed measurement may get closer to a signal intensity expected from a total copy number of two rather than from one copy. Even though there might be a considerable degree of likelihood of the observed measurement deriving from a single copy, one might discard this possibility and errantly conclude two copies were present. Since previous tools cannot handle multiple possibilities with likelihood values, the use of wrongly determined total copy numbers as the input would lead to incorrect haplotype inference.

To overcome this problem, we developed an algorithm that handles such likelihood values for CNV haplotype inference. This algorithm is designed as a post-process to be performed after a calling method, such as Birdsuite (Korn *et al.* 2008), determines the boundaries of CNVs and computes the parameters of a probabilistic model that determines the most probable total copy numbers or unphased genotypes in its framework. Likelihood values as inputs of our algorithm are given through such a probabilistic model. We implemented this algorithm in a computational tool called *CNVphaserPro* (CNV phasing tool using probabilistic information). This tool can infer haplotypes composed of not only integer copy numbers (ICNs) but also single nucleotide variations in a CNV (SNVCs) (Kato *et al.* 2008a) and SNPs, whose relationship is illustrated in Figure 1. As a by-product, this tool can also estimate individual’s diplotypes. We performed simulation studies based on known haplotypes and real microarray noise, demonstrating that this tool successfully inferred haplotypes and diplotypes from noisy microarray data, and that the tool even had an ability to correct total copy numbers and unphased genotypes that are wrongly determined due to noise. Encouraged by the success in simulation studies, we applied the tool to real microarray data for individuals of European descent from Utah (CEU) and obtained the estimation of haplotype frequencies and individuals’ diplotypes in 1486 CNV regions along the human genome.

**Figure 1:**
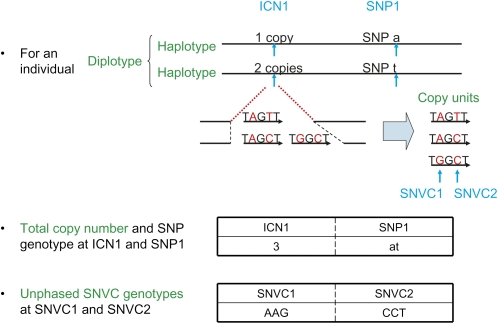
Illustration of ICN, SNP, and SNVC sites. (Upper part) “ICN”, “SNP”, and “SNVC” represent integer copy number, single nucleotide polymorphism, and single nucleotide variation in a CNV, respectively. A “copy unit” represents the unit of DNA sequence that is duplicated in a CNV region (Kato *et al.* 2008a). In this illustration, most invariant bases in copy units are omitted for the purpose of simplicity. Lower part: High-throughput experimental technologies give data on the total number of copies over a diplotype or an unphased sequence genotype.

## Materials and Methods

### The algorithm

The aim of the algorithm is to estimate the frequencies of haplotypes from a group of total copy numbers and/or unphased genotypes with likelihood values for randomly sampled individuals. Here we call a total copy number the total number of allelic copies over a diplotype (a pair of haplotypes) at an ICN site. For example, allelic copy number 1 in one haplotype and allelic copy number 2 in the other haplotype at an ICN site result in the total copy number of 3. We call an unphased genotype a nucleotide sequence for which the phase is unknown but the total number of each allelic nucleotide base over a diplotype at an SNVC site is known. For example, regarding the unphased genotype AAG at an SNVC site, the phase is unknown (it may be A in one haplotype and both A and G in the other haplotype, or both A and A in one haplotype and G in the other haplotype, or another) but the total numbers of the alleles A and G are known as two and one, respectively (Figure 1).

For that aim, we combined an algorithm of Kang *et al.* (Kang *et al.* 2004) with the algorithms of CNVphaser (Kato *et al.* 2008a) and MOCSphaser (Kato *et al.* 2008b) so that the proposed algorithm could incorporate likelihood values for total copy numbers and unphased genotypes into the framework of the expectation-maximization (EM) algorithm for CNV phasing. The algorithm by Kang *et al.*, which is called the GenoSpectrum-EM algorithm, uses the EM algorithm that considers likelihood values for SNP genotypes to infer SNP haplotypes, not CNV haplotypes. CNVphaser and MOCSphaser use the EM algorithm to infer CNV haplotypes from total copy numbers and unphased SNVC genotypes, but do not consider likelihood values for total copy numbers or unphased genotypes.

The proposed EM algorithm can be divided into two procedures: enumeration and iterative calculation. In the initial procedure, we enumerate all possible diplotypes that are consistent with total copy numbers or unphased genotypes across multiple sites for every individual. More specifically, we first enumerate all possible diplotypes per site. For example, if a total copy number at an ICN site is 3, all possible diplotypes we enumerate are [0/3] and [1/2], where a slash (/) represents the separator between haplotypes (we do not distinguish the order of haplotypes). Similarly, if an unphased genotype at an SNVC site is AAG, all possible diplotypes are [-/A, A, G], [A, A/G], and [A/A, G], where a dash (-) represents a deletion and a comma (,) represents the separator between copy units (we do not distinguish the order of copy units in a haplotype). Here, we call a copy unit the unit of DNA sequence that is duplicated in a CNV region (Figure 1). Then, we enumerate all possible diplotypes across multiple sites. For example, if we have the one-site diplotypes [0/3] and [1/2] at an ICN site, and the one-site diplotype [a/t] at an SNP site, all the possible diplotypes across these two sites are [0_a/3_t], [0_t/3_a], [1_a/2_t], and [1_t/2_a], where an underscore (_) represents the separator between sites.

In this procedure, we also calculate a likelihood across multiple sites for each diplotype, following the assumption of Kang *et al.* (2004) that the occurrence of an experimental measurement, such as a signal intensity, at one site given a diplotype is independent of that at another site (also see Equations 1 and 6 below). Note that this assumption does not require linkage equilibrium – copy numbers or genotypes at multiple sites are independent of each other. Also note that, for microarray probes that are determined to be in the same state by a segmentation tool, it might be necessary to aggregate the correlated measurement values (*e.g.*, through median) into one value to treat the probe locations as one site.

Under the assumption, the likelihood *λ_j,k_* is calculated as follows:

$$\lambda_{j,k}=\mathrm{Pr}(x_{j,k}|d_{j,k})=\prod_{s=1}^{S}\mathrm{Pr}(x_{j,k,s}|d_{j,k,s})$$ (1)

where *k* denotes the index for the diplotype of individual *j*, and *s* and *S* denote the index for a site and the total number of sites in a given dataset, respectively. The variable **x***_j,k_* denotes the observed measurements such as the signal intensities or the log ratio intensities across multiple sites, and *x_j,k,s_* denotes the observed measurement at site *s* in **x***_j,k_*. The symbol *d_j,k_* denotes the diplotype indexed by *k* for individual *j* across multiple sites, and *d_j,k,s_* denotes the one-site diplotype at site *s* in *d_j,k_*. The last term Pr(*x_j,k,s_* | *d_j,k,s_*) is equal to the probability of *x_j,k,s_* given the total copy number or unphased genotype of diplotype *d_j,k,s_* (because we do not distinguish the signal intensities of, for example, [0/3] and [1/2]), and this probability is calculated from a statistical model of signal intensities or log ratio intensities in microarrays. The choice of a statistical model and a method to estimate its parameters is left to a user. For example, in simulation studies, we used those of Birdsuite (Korn *et al.* 2008), which employs a Gaussian mixture model and parameter estimation by the expectation-maximization algorithm. We obtained the last term probability, or likelihood, directly from the probability density of a signal intensity in a normal distribution of the model.

After enumeration, we perform the iterative calculation, in which we iteratively repeat the expectation (E) and the maximization (M) steps. In the E step, we calculate the proportion of the frequency of a diplotype to the sum of the frequencies of all diplotypes in an individual, using likelihood *λ_j,k_*. The equation at the E step is:

$$w_{j,k}=\frac{\lambda_{j,k}P(d_{j,k})}{\sum_{k=1}^{D_{j}}\lambda_{j,k}P(d_{j,k})}$$ (2)

where *w_j,k_* denotes the diplotype proportion, *D_j_* denotes the number of possible diplotypes of individual *j*, and *P* denotes the population frequency. *P*(*d_j,k_*) is calculated from Hardy-Weinberg equilibrium (HWE):

$$P(d_{j,k})=P(h_{l}\text{ / }h_{m})=\{\begin{array}{l} P(h_{l})P(h_{m})\text{ if }l=m\\ 2P(h_{l})P(h_{m})\text{ if }l\neq m \end{array}$$ (3)

where the diplotype *d_j,k_* consists of the haplotypes indexed by *l* and *m* (*h_l_* and *h_m_*). At the M step, the frequency of a haplotype is calculated from the number of the haplotype in consideration of the diplotype proportion calculated at the E step. The equation at the M step is:

$$P(h_{i})=\frac{\sum_{j=1}^{N}\sum_{k=1}^{D_{j}}\delta (h_{i},d_{j,k})\cdot w_{j,k}}{2N}$$ (4)

$$\delta (h_{i},d_{j,k})=\{\begin{array}{l} 2\text{ if }d_{j,k}\text{ includes two }h_{i}\\ 1\text{ if }d_{j,k}\text{ includes one }h_{i}\\ 0\text{ if }d_{j,k}\text{ includes no }h_{i} \end{array}$$ (5)

where *N* denotes the number of individuals in the dataset. After this M step, the iteration goes back to the E step to update the diplotype proportion, and in turn goes to the M step to update the haplotype frequency until the log-likelihood converges. The log-likelihood ln *L* is:

$$\ln L=\ln\prod_{j=1}^{N}\left(\sum_{k=1}^{D_{j}}\mathrm{Pr}(x_{j,k}|d_{j,k})\cdot \mathrm{Pr}(d_{j,k}|\theta )\right)=\ln\prod_{j=1}^{N}\left(\sum_{k=1}^{D_{j}}\lambda_{j,k}P(d_{j,k})\right)$$ (6)

where **θ** denotes (*P*(*h_1_*), *P*(*h_2_*), ...).

The main difference of this EM algorithm from those of CNVphaser (Kato *et al.* 2008a) and MOCSphaser (Kato *et al.* 2008b) is the inclusion of the likelihood term *λ_j,k_* in equations 2 and 6. By incorporating this term, total copy numbers and/or unphased genotypes are probabilistically represented. Equations 3, 4, and 5 are the same. The main difference of our algorithm from the GenoSpectrum-EM algorithm of Kang *et al.* (Kang *et al.* 2004) lies in the enumeration procedure, in which our algorithm can enumerate diplotypes composed of any combination of ICNs, SNVCs, and SNPs, whereas Kang *et al.*’s algorithm enumerates diplotypes composed only of SNPs. The equations in our EM algorithm are essentially the same as those in Kang *et al.*’s EM algorithm.

### Software

We implemented this algorithm into a computational tool called CNVphaserPro, which is available online (http://rulai.cshl.edu/people/kato/). The advancements of this tool from the previous tools (Kato *et al.* 2008a; Kato *et al.* 2008b) are shown in the supporting information, Table S1. The tool can handle missing calls and use multiple sets of randomly-generated initial values for multiple EM runs.

## Results

### Simulation tests

**Figure 2:**
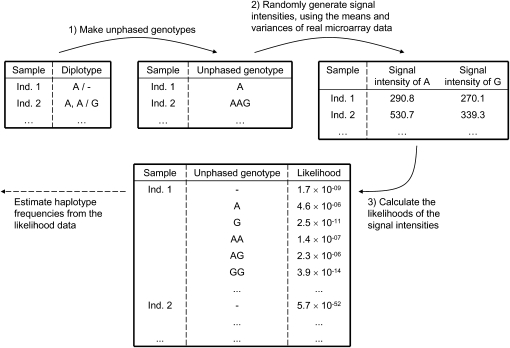
Illustration of simulation. “Ind.” is short for individuals. The symbols “-”, “/”, and “,” in the top left table represent a deletion, the separator between haplotypes, and the separator between copy units in a duplication. (1) Using known diplotypes (Sachse *et al.* 1997), we made unphased genotypes. (2) We randomly generated signal intensities for the unphased genotypes, using normal distributions with the means and variances taken from real microarray data (Korn *et al.* 2008). (3) We calculated the likelihoods of the signal intensities for all possible unphased genotypes (or total copy numbers), based on the normal distributions above.

We tested whether the algorithm could correctly restore haplotype frequencies in simulated datasets, which were made as follows (Figure 2). We first obtained unphased genotypes from the known haplotypes (listed in File S1) of 588 individuals at 14 sites (Sachse *et al.* 1997), and then randomly generated signal intensities for the unphased genotypes at each site, using two-dimensional normal distributions for the genotypes. Their means and variances were derived from real microarray data (Korn *et al.* 2008). Next, we used these normal distributions to calculate the probability densities of the signal intensities for all possible unphased genotypes and used them as likelihood values. For total copy numbers, we took the summation of the likelihood values across unphased genotypes with the same copy number to obtain the likelihood values of total copy numbers. See File S2 for details on generating the simulation data. We input the likelihood values separately at each site into CNVphaserPro for one-site inference. We used the known haplotypes directly as the answer haplotypes.

We first show an example of our results on one-site ICN inference (Table 1). The estimated frequencies were all close to the answer frequencies; hence, we concluded that the algorithm succeeds in the estimation. For comparison, we estimated haplotype frequencies by the previous algorithm [of MOCSphaser (Kato *et al.* 2008b) and CNVphaser (Kato *et al.* 2008a)], using the most likely total copy numbers as the input, which were defined as the one with the largest likelihood value of all possible total copy numbers. As demonstrated previously (Excoffier and Slatkin 1995; Kato *et al.* 2008a; Kato *et al.* 2008b), we quantified the degree of estimation error by the total variation distance *TV*:

$$TV=(1/2)\sum_{i}\left|p_{i}-{\hat{p}}_{i}\right|$$ (7)

where $p_{i}$ and ${\hat{p}}_{i}$ are the true and estimated frequencies of the haplotype *i*. As a result, the estimation error of the current algorithm was much less than that of the previous algorithm (the error rate 2% *vs.* 12%) (Table 2). When we compared the most likely total copy numbers with the answer total copy numbers, 22% were incorrect, on average. This indicates that total copy numbers determined with the largest likelihood value are often incorrect due to microarray noise, which would result in the poorer estimates by the previous algorithm.

**Table 1:** Examples of haplotype frequency estimation

Variation Type	Haplotype*a*	Known Frequency	Estimated Frequency
One ICN site	1 copy	0.9609	0.9607
	0 copies	0.0196	0.0199
	2 copies	0.0196	0.0194
	3 copies	*NA*	1.0 × 10^−10^
	—	—	—
One SNVC site	A	0.9600	0.9598
	—	0.0196	0.0199
	A, A	0.0196	0.0194
	B	0.0009	0.0009
	A, B	*NA*	2.7 × 10^−10^
	—	—	—
Two SNVC sites	-A	0.9592	0.9589
	–	0.0196	0.0199
	-A, -A	0.0196	0.0194
	-B	0.0009	0.0009
	AA	0.0009	0.0009
	BA	*NA*	3.6 × 10^−5^
	—	—	—

*NA* indicates that the corresponding haplotypes did not exist in the known dataset.

^a^ The symbols A and B represent different nucleotide bases, and “-” and “,” represent a deletion and the separator between copies in a duplicated region, respectively. We omitted haplotypes with estimated frequencies of less than 10^−10^ (all such haplotypes did not exist in the known dataset).

**Table 2:** Error rate of haplotype frequencies estimated by the current and previous algorithms

Variation Type	*TV* for the Current Algorithm	*TV* for the Previous Algorithm
One ICN site	0.019 ± 0.036	0.123 ± 0.122
One SNVC site	0.008 ± 0.010	0.118 ± 0.117
Two SNVC sites	0.022 ± 0.029	0.176 ± 0.109

*TV* measures the deviation of estimated frequencies from answer frequencies. The numbers are the mean ± SD across the 14 sets.

We also inferred individuals’ diplotypes of ICNs by selecting the diplotype with the largest diplotype proportion value in equation 2 (we did not use a threshold), and then evaluated inference accuracy by the proportion of inferred diplotypes that were the same as the answers. The result showed a good accuracy, 95% on average (Table 3). We also used this proportion as the rate of total copy numbers correctly inferred by the current algorithm, because total copy numbers can be obtained simply from diplotypes. Thus, we compared this rate with the rate of correct ones of the most likely total copy numbers. As a result, the former rate (95%) was clearly larger than the latter rate (78%) (Table 3), which indicates that the current algorithm has an ability to correct total copy numbers incorrectly determined with the largest likelihood value. In fact, there were total copy numbers that were incorrectly determined with the largest likelihood value but were correctly inferred by the current algorithm; though we did find some, albeit far fewer, of the opposite case (Table 3).

**Table 3:** Inference accuracy of individuals’ diplotypes and total copy numbers or unphased genotypes

Variation Type	Accuracy of Diplotypes by the Current Algorithm*a*	Accuracy of the Most Likely Unphased Copy Numbers or Genotypes*b*	Correction*c*	Corruption*d*
One ICN site	94.9 ± 5.4%	78.1 ± 20.6%	19.2 ± 17.9%	2.5 ± 2.9%
One SNVC site	95.6 ± 4.3%	78.6 ± 20.0%	19.1 ± 17.7%	2.2 ± 2.3%
Two SNVC sites	94.9 ± 6.1%	66.0 ± 17.4%	29.4 ± 14.7%	0.5 ± 0.4%

^a^ Proportion of diplotypes (and also total copy numbers or unphased genotypes) that were correctly inferred by the current algorithm. The numbers are the mean ± SD across the 14 sets.

^b^ Proportion of correct ones of the most likely total copy numbers or unphased genotypes, which have the largest likelihood value of all possible total copy numbers or unphased genotypes and therefore we used as the input of the previous algorithm. As with diplotypes, we required that correct ones should agree with answers over all sites. The numbers are the mean ± SD across the 14 sets.

^c^ Proportion of total copy numbers or unphased genotypes that were incorrectly determined with the largest likelihood value but were correctly inferred by the current algorithm. The numbers are the mean ± SD across the 14 sets.

^d^ Proportion of total copy numbers or unphased genotypes that were correctly determined with the largest likelihood value but were incorrectly inferred by the current algorithm. The numbers are the mean ± SD across the 14 sets.

We also estimated the frequencies of haplotypes at one- and two-SNVC sites. In the two-site case, we chose pairs out of the 14 sites randomly a total of 14 times and input simulated likelihood values at the pairs into our algorithm. We show examples of results in both cases (Table 1), which demonstrates that the estimated frequencies were almost the same as the answer frequencies. The *TV* index revealed that the estimation error of the current algorithm was much less than that of the previous algorithm (the error rate 1–2% *vs.* 12–18%) (Table 2). Regarding the inference of individuals’ diplotypes, the inference results also showed a good accuracy: on average, 95–96% were correct in both cases (Table 3). As with ICNs, the current algorithm corrected unphased genotypes that were incorrectly determined with the largest likelihood value (Table 3). Most importantly, improvement by the current algorithm from the previous algorithm was greater in the case of two SNVC sites, which is more complicated than the single ICN and SNVC cases.

### Influence of sample size

We examined the influence of sample size on algorithm performance. For that purpose, we used population frequencies calculated from the known haplotypes of the 588 individuals to randomly sample haplotypes, and then randomly paired them on the basis of HWE to make answer datasets with different simple sizes. We made 10 replicate answer sets for each simple size. For each answer set, we used the same procedures as in the first simulation to make an input dataset.

**Figure 3:**
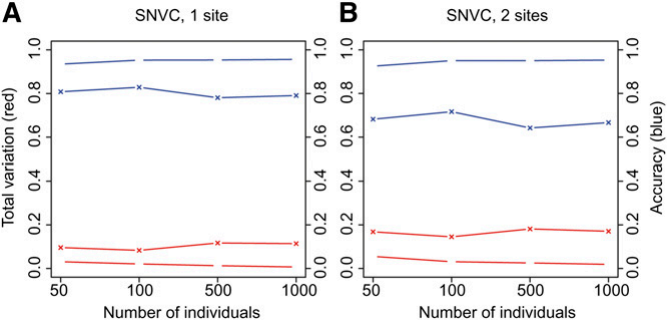
Influence of sample size on performance. The left *y*-axis represents the deviation (the *TV* index) of estimated haplotype frequencies from answer frequencies for the current (red line with circles) and the previous (red line with crosses) algorithms. The right *y*-axis represents the proportion of diplotypes correctly inferred by the current algorithm (blue line with circles) or unphased genotypes correctly determined with the largest likelihood value (blue line with crosses). Points in the *y*-axes at each sample size indicate the mean of values over 10 different answer sets × 14 different site sets. (A) For one SNVC site. (B) For two SNVC sites. The result of one ICN site is not shown, because this result was similar to that of one SNVC site.

As a result, with the increase of the sample size, the current algorithm showed a slight but constant improvement in both the *TV* performance and the accuracy of inferred genotypes (Figure 3) or inferred copy numbers (data not shown), though the values of both indices were nearly saturated. In contrast, the previous procedures (the previous algorithm and the method to determine unphased copy numbers or genotypes by the largest likelihood value) did not always show a constant improvement. The current algorithm showed a better *TV* performance and a better genotype or copy-number inference at all sample sizes as compared to the previous procedures. In the two-SNVC case, the current algorithm showed the largest difference in *TV* and the accuracy from the previous procedures. In all the cases, the accuracy of diplotypes inferred by the current algorithm was greater than 90% even at a sample size of 50 and greater than 95% at a sample size of 100 or more. The *TV* values were extremely low at any sample size in all the cases (6% at a sample size of 50 at two-SNVC sites; less than 4%, otherwise).

### Real data application

We applied the phasing tool to real data taken with NimbleGen HD2 comparative genomic hybridization (CGH) arrays for the HapMap Phase3 CEU population. We identified 1486 CNV regions for 106 unrelated individuals, and then used the median log-ratio intensities across probes present in the intersections of CNV segments to obtain their likelihood values for the total copy numbers of zero to four, based on the Gaussian mixture model. See File S3 for details on processing the real data. We next applied the phasing tool to the likelihood values and estimated haplotype frequencies (File S4) and diplotypes (File S5) in the CNV regions along the genome. We compared the predicted diplotypes (with the diplotype proportions > 0.98) of two individuals to deletions that were extensively detected by next-generation sequencing at a high coverage (42×) for the same individuals (Mills *et al.* 2011). We found that eight out of the nine of our deletions were consistent with theirs and only 3.3% (65/1983) out of our 1/1 diplotypes were inconsistent.

**Figure 4:**
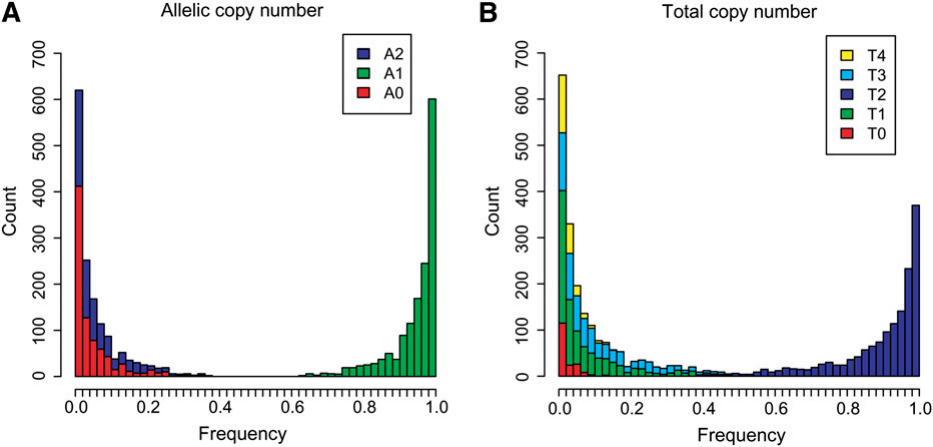
Frequency spectrums. The width of each bin is 2%. Alleles with a very small [<1 / (2 × the number of individuals)] or large [>1 − 1 / (2 × the number of individuals)] frequency are excluded from the counts. (A) The frequency spectrum of alleles. A*n* (*n* = 0, 1, and 2) in the box represents an allelic copy number. (B) The frequency spectrum of total copy numbers derived from the allelic copy numbers. T*n* (*n* = 0, 1, ..., 4) in the box represents an total copy number.

Using all the CNV regions, we drew an allele frequency spectrum (Figure 4)—a basic graph that summarizes their population-genetic nature. The graph shows that the zero-copy (A0) and two-copy (A2) alleles tended to have small frequencies and that the number of the zero-copy allele was somewhat larger than that of the two-copy allele. We found 15 CNV regions where both zero-copy and two-copy alleles had the population frequencies of more than 1%, which indicates that these are tri-allelic. Twelve out of the fifteen were overlapped with CNVs in the Database of Genomic Variants (Zhang *et al.* 2006) and eight out of the twelve had been defined as multi-allelic CNVs (having both gains and losses) in CEU or a French population (Conrad *et al.* 2010; De Smith *et al.* 2007). We show examples of such CNV regions overlapped with genes (Table 4). The CNVs overlapped with *ACOT11* and *CWF19L2* were previously reported as multi-allelic in CEU (Conrad *et al.* 2010), but the CNV overlapped with *EYA2* was not reported. A homolog to *ACOT11* in mouse has been associated with obesity, but the function of *CWF19L2* is unknown. *EYA2* may play a role in eye development.

**Table 4:** Examples of CNV regions that overlapped with genes and that had substantial amounts of estimated frequencies for both zero-copy and two-copy alleles

Chromosome	From	To	Haplotype*a*	Frequency	Overlapping Gene	Synopsis of the Function
1	54,858,310	54,866,895	0 copies	0.025	*ACOT11*	Associated with obesity in mouse
			1 copy	0.920		
			2 copies	0.055		
20	45,247,180	45,253,878	0 copies	0.073	*EYA2*	Possible involvement in eye development
			1 copy	0.905		
			2 copies	0.023		
11	106,745,435	106,757,969	0 copies	0.038	*CWF19L2*	Unknown (*CWF19*-like 2, cell cycle control in *S. pombe*)
			1 copy	0.696		
			2 copies	0.266		

The “Chromosome”, “From”, and “To” columns indicate the chromosomal locations of CNV regions (hg18).

^a^ We omitted haplotypes with estimated frequencies of less than 10^−3^.

## Discussion

We used simulated datasets that were as close to real haplotypes and microarray noise levels as possible, and we demonstrated successful estimation by our algorithm. The reason for this success is the use of likelihoods to quantify fluctuations caused by noise. For example, even when a signal intensity is observed far away from the center of a distribution of the original total copy number, our algorithm does not discard the possibility that the signal came from this copy number, but considers the signal as a rare outcome with a small likelihood value. The usefulness of this strategy of incorporating genotype likelihoods (instead of called genotypes) into haplotype frequency estimation was demonstrated in SNP cases (Browning and Yu 2009; Kang *et al.* 2004; Yu *et al.* 2009). For example, in one recent study using BEAGLE version 3.1 (Browning and Yu 2009), this strategy was incorporated into SNP haplotype phasing based on an hidden Markov model (HMM). In our study, combining likelihoods with the EM algorithm based on a multinomial model (Excoffier and Slatkin 1995), we demonstrated its usefulness in CNV haplotype inference.

As a by-product, our algorithm also inferred individuals’ diplotypes. The accuracy of the diplotype inference went over 90% even at a sample size of 50 and over 95% at a sample size of 100 or more, which indicates that even a sample size around 100 is enough for a good inference in typical cases. By accurately inferring diplotypes, the algorithm even corrected total copy numbers or unphased genotypes that were incorrectly determined with the largest likelihood value. Substantial amounts of 21–34% incorrect rates were decreased to only 4–5% through correction by the current algorithm.

Most importantly, as the sample size increased, the estimation error index *TV* and the accuracy of estimated total copy numbers or unphased genotypes were improved in the current algorithm, but not always in the previous procedures. This is because there are always incorrect unphased copy numbers or genotypes determined with the largest likelihood value regardless of increasing a sample size (as shown by the blue lines with cross in Figure 3), as long as signal intensities coming from different unphased copy numbers or genotypes are not clearly separated with each other. Thus, increasing a sample size does not always improve estimation accuracy in the previous algorithm, but does so in the current algorithm—this is another advantage of the current algorithm.

There would be several algorithmic issues to be addressed in the future. Our algorithm assumes HWE; hence, if a population does not satisfy HWE, the estimation would be worse. Regarding the EM algorithm used in SNP haplotype inference, it is known that deviations from HWE do not greatly impact the accuracy (Niu *et al.* 2002). In addition, a large-scale CNV study has found that most CNVs (98% of the bi-allelic CNVs) are in HWE (McCarroll *et al.* 2008). Furthermore, in the first type of simulation, we demonstrated accurate estimation by our algorithm for known haplotypes in a natural population, in which HWE was not artificially introduced. Nevertheless, it is necessary to know how much impact deviations from HWE have on the accuracy.

Another issue is to handle haplotypes composed of a number of sites. Currently, to analyze haplotypes composed of a few sites meets the needs for practical applications in CNV research, because urgent issues in this field are, first, association studies of CNVs, where current studies handle CNV regions as single sites (loci) and perform statistical tests separately per region (Stefansson *et al.* 2008; The International Schizophrenia Consortium 2008), and second, to find CNVs tagged by SNPs, where two-site haplotypes composed of an ICN and a SNP sites are phased to calculate a two-site LD index such as *R^2^* (Conrad *et al.* 2010; Kato *et al.* 2010; McCarroll *et al.* 2008). Our algorithm can fulfill these needs. In fact, our algorithm finished calculations for one and two sites across 588 samples within a short time (13 and 187 sec on average for one and two sites, respectively) on our machine with eight Xeon 2-GHz CPUs and 8 GB RAM, and also for three sites within a reasonable time (49 min on average). In addition, the algorithm inferred allelic copy numbers in all the 1486 CNV regions in the real data application within 18 hr on the same machine. However, our EM-based algorithm may not be suitable for applications beyond the current needs, since it takes significant time to handle a large number of sites due to the exhaustive enumeration. For example, our algorithm did not complete calculations within a reasonable time (24 hr) for four sites in some datasets, though the computational time can be reduced to some degree by cutting off states with the likelihood value of zero or nearly zero (after normalizing likelihood values, since only the relative values are meaningful).

For applications beyond the current urgent needs, the algorithm will have to be improved. For example, recently the GrEM algorithm (Shindo *et al.* 2009) and the algorithm implemented in polyHap v2.0 (Su *et al.* 2010), which uses an HMM, have been proposed to deal with a larger number of sites for CNV haplotype inference from data unambiguously determined. They could be extended or modified to accommodate data with noise. In addition, a computational tool called cnvHap has recently been developed in order to call (unphased) copy numbers or sequence genotypes based on a haplotype HMM (Coin *et al.* 2010). Although the aim of cnvHap is not to estimate haplotype frequencies, information on haplotypes might be extracted from this approach. Other possible approaches include partition-ligation EM (Kato *et al.* 2008a; Qin *et al.* 2002), Gibbs sampling, and coalescent-based sampling (Niu 2004).

We applied our tool to real ICN data. Although we demonstrated accurate inference of SNVC haplotypes in simulation studies, it will be worth trying real SNVC data when such data in a large sample size (>100 individuals) are accessible. Regarding association studies of CNVs, current studies using microarrays focus on ICNs or ICN groups known as gains (>2 copies) and losses (<2 copies). Recently, a theoretical study examined statistical power in association studies based on ICN haplotypes (Ohashi 2009). Because SNVCs, which are a form of variation mixed of SNPs and ICNs, are more likely to have a phenotypic effect than only SNPs or only ICNs (Hosono *et al.* 2008; Kato *et al.* 2008a), it would be important that these studies are extended for SNVC haplotypes, in which case, our algorithm will be of essential use.

Because microarrays are currently less costly than next-generation sequencing in the same sample size in genome-wide CNV studies, our haplotype phasing based on microarray data are cost effective for rapidly investigating hundreds or thousands of individuals. Recently, a study has developed a successful method to estimate total copy numbers from read depth in next-generation sequencing and applied it to three individuals (Alkan *et al.* 2009). Taking account of the fact that read counts fluctuate by noise (Yoon *et al.* 2009), our likelihood method would be applicable also for such copy numbers when sample size is sufficiently obtained in the future.

## Supplementary Material

Supporting Information
