# Supplementary material for: Inferring Haplotypes of Copy Number Variations From High-Throughput Data With Uncertainty
Source: G3 (Bethesda). 2011 Jun 1;1(1):35–42. doi: 10.1534/g3.111.000174 (PMC3276117; doi:10.1534/g3.111.000174)
Supplement: Supporting Information [file supp_1_1_35__index.html]

Supporting Information 

# Inferring Haplotypes of Copy Number Variations From High-Throughput Data With Uncertainty

## Supporting Information for Kato *et al.*, 2011

**Files in this Data Supplement:**

- Supporting Information - Files S1-S5 and Table S1 (PDF, 272 KB)
- Table S1 - Functional comparison with previous phasing tools (PDF, 40 KB)
- File S2 - Details on generating simulation data (PDF, 48 KB)
- File S3 - Details on processing real data (PDF, 52 KB)
- File S1 - Known haplotypes used in simulation tests (.zip, 4 KB)
- File S4 - Estimated haplotype frequencies in CNV regions along the human genome (.zip, 120 KB)
- File S5 - Estimated individuals� diplotypes in CNV regions along the human genome (.zip, 1.8 MB)
