# Supplementary material for: Inferring Haplotypes of Copy Number Variations From High-Throughput Data With Uncertainty
Source: G3 (Bethesda). 2011 Jun 1;1(1):35–42. doi: 10.1534/g3.111.000174 (PMC3276117; doi:10.1534/g3.111.000174)
Supplement: Supporting Information [file supp_1.1.35_000174SI.pdf]

Table S1 Functional comparison with previous phasing tools

	Variation type[a]			Representation type[b]	
	ICN	SNVC	SNP	Logical representation	Likelihood representation
MOCSphaser	**Yes**	No	**Yes**	**Yes**	No
CNVphaser	Only 1 site	**Yes**	No	No	No
CNVphaserPro	**Yes**	**Yes**	**Yes**	**Yes**	**Yes**

[a] "Yes" means that a tool can handle not only one site but also multiple sites.

[b] "Logical representation" means that ICNs can be expressed as or-type and greater-type copy numbers (KATO *et al.* 2008) such as "2 OR 3 copies" and ">4 copies." The most characteristic point of CNVphaserPro is the ability to handle likelihood representation.

File S1

Known haplotypes used in simulation tests

These known haplotypes (phased diplotypes) are obtained from Sachse et al (SACHSE *et al.* 1997). The symbols "-", ",", and "/" represent a deletion, the separator between copy units in a duplication, and the separator between haplotypes, respectively. Of 1176 (588 × 2) haplotypes, there are 13 distinct haplotypes including haplotypes with two-copy duplications, a haplotype in which nucleotide bases are deleted entirely across 14 sites, and haplotypes in which nucleotide bases are deleted at some of the 14 sites.

File S1 is available for download as a text file at http://www.g3journal.org/lookup/suppl/doi:10.1534/g3.111.000174/-/DC1/FileS1.zip.

File S2

Details on generating simulation data

We made simulated datasets from known real haplotypes of Sachse et al. (Sachse *et al.* 1997), who closely performed a series of target-specific experiments (long template PCR, nested PCR-RFLP method, RFLP-Southern blotting, and allele-specific PCR) to obtain diplotypes at 14 SNVC sites in the *CYP2D6* gene for 588 individuals of a Caucasian population. To our knowledge, this is the only known haplotype set that is composed of a large number of well-characterized haplotypes.

To make an input simulated dataset, we first counted up the number of each allele over a known diplotype at each of the 14 sites for each of the 588 individuals and made an unphased genotype (*e.g.*, diplotype [A, B/A] resulted in unphased genotype AAB). Next, for each of the unphased genotypes, we used a 2-dimensional normal distribution as an error model to randomly generate signal intensities. The mean and variance of a 2-dimensional normal distribution were obtained from real experimental data measured in the Affymetrix SNP 6.0 array that were downloaded from the Birdsuite website (Korn *et al.* 2008). Birdsuite regards microarray signal intensities of two alleles (represented as "A" and "B") as outcomes from the 2-dimensional normal distributions for different unphased genotypes and outputs the means and variances of such normal distributions on the basis of the Gaussian mixture model. From the downloaded data, we randomly picked up 14 sites each of which had the calculated means and variances of multiple normal distributions that corresponded to "-" (deletion), A, B, AA, AB, BB, AAA, AAB, ABB, BBB, AAAA, AAAB, ..., and BBBB, and we assigned each of these 14 sites to each of the known 14 SNVC sites (because there were no CNV sites overlapped with the *CYP2D6* gene in the downloaded data). Then, we used an unphased genotype at an SNVC site and also the normal distribution for the same genotype to randomly generate signal intensities (*e.g.*, we used both unphased genotype AAB and 2-dimenstional normal distribution for AAB to generate random signal intensities of 447.2 for "A" and 222.7 for "B"). We used the "mvtnorm" library in R for this calculation.

Finally, we calculated the probability densities of signal intensities using the normal distributions for all possible unphased genotypes, and used them as likelihood values (*e.g.*, a likelihood value for which signal intensities 447.2 and 222.7 for "A" and "B" came from AAB was 5.5×10^{-7}, that from AAA was 1.8×10^{-5}, and *etc.*). Thus, we made an input dataset in which a likelihood value was assigned to each unphased genotype at each site for each individual.

We also made ICN datasets from the SNVC datasets. As the answer dataset, we just counted the number of copies at each of the 14 sites to make diplotypes represented in ICN for each of the 588 individuals (*e.g.*, diplotype [A, B/A] resulted in ICN diplotype [2 copies/1 copy]). As an input dataset, we summed up allelic copy numbers to obtain total copy numbers (*e.g.*, diplotype [2 copies/1 copy] resulted in total copy number 3). To obtain likelihood values for total copy numbers, we summed up the likelihood values calculated for unphased genotypes across all unphased genotypes that had the same copy number (*e.g.*, for total copy number 3, we summed up likelihood values across AAA, AAB, ABB, and BBB since these have three copies) because events to observe such unphased genotypes are mutually exclusive. Thus, we obtained a likelihood value assigned to each total copy number at each site for each individual.

File S3

Details on processing real data

For real data application, we used data collected using NimbleGen HD2 comparative genomic hybridization (CGH) array platform in the HapMap Phase3 CEU population. The data processing steps of scanning and spatial-normalization were performed in NimbleGen and the data processes of normalization between samples and GC-content correction of log2 ratios were performed at Cold Spring Harbor Laboratory. The details on the data processes are described in the literature (McCarthy *et al.* 2009). Following the previous procedure (Fridlyand *et al.* 2004; Kato *et al.* 2010; Komura *et al.* 2006), we first performed CNV segmentation and then used the median of log ratio intensities over probes in a segment for total copy numbers.

Specifically, we defined CNV segments only when CNV segments identified by two different segmentation tools (Day *et al.* 2007; Pique-Regi *et al.* 2008) were overlapped, more than six probes were included in segments, all the probes in a segment were mapped to only one chromosomal position, and the segment lengths were between 1 kb and 1 Mb. Also, we excluded some individuals with too many or too few segments (by outlier analysis: more/less than 3 × |third/first quartile – median|) to minimize individuals possibly affected by hybridization errors. Then, we selected CNV regions in each of which no individuals had multiple segments, the number of chromosomal parts with the maximum population frequency of CNV segments (*i.e.*, core fragment-site (Kato *et al.* 2010)) was only one, and the core part covered more than three fourths of the region length so that we could limit to regions with simple segment patterns (in our experience, segment patterns in a region tend to become complicated when the reference individual in array CGH, not test individuals, has CNVs). We selected the autosomal chromosomes' CNV regions where the core parts had more than five probes, and took the median of log ratio intensities over those probes for each individual both with and without CNV segments.

Next, we used the Gaussian mixture model and parameter estimation by the expectation-maximization method to calculate its means and variances, employing the "mclust" library in R. Then, we calculated the probability densities of the median log-ratio intensities for the multiple Gaussian distributions corresponding to zero to four copies to obtain likelihood values. For haplotype inference, we only used unrelated individuals (*i.e.*, parents).

File S4

Estimated haplotype frequencies in CNV regions along the human genome

We excluded CNV regions that were likely to be monomorphic (the frequency of the one-copy allele was more than 1 – 1 / (2 × the number of individuals)), and CNV regions in which the reference individual was likely to have a CNV (that frequency was less than 0.6). The first column indicates the ID of the core site in each CNV region. The chromosomal positions are of hg18 (NCBI Build 36). "Confidence" means the mean of the diplotype proportions of the most probable diplotypes across individuals for a region.

File S4 is available for download as a text file at http://www.g3journal.org/lookup/suppl/doi:10.1534/g3.111.000174/-/DC1/FileS4.zip.

File S5

Estimated individuals' diplotypes in CNV regions along the human genome

We used the same CNV regions as in File S4.

File S5 is available for download as a text file at http://www.g3journal.org/lookup/suppl/doi:10.1534/g3.111.000174/-/DC1/FileS5.zip.

LITERATURE CITED

DAY, N., A. HEMMAPLARDH, R. E. THURMAN, J. A. STAMATOYANNOPOULOS and W. S. NOBLE, 2007 Unsupervised segmentation of continuous genomic data. Bioinformatics **23:** 1424-1426.

FRIDLYAND, J., A. M. SNIJDERS, D. PINKEL, D. G. ALBERTSON and A. N. JAIN, 2004 Hidden Markov models approach to the analysis of array CGH data. J. Multivariate Anal. **90:** 132-153.

KATO, M., T. KAWAGUCHI, S. ISHIKAWA, T. UMEDA, R. NAKAMICHI *et al.*, 2010 Population-genetic nature of copy number variations in the human genome. Hum. Mol. Genet. **19:** 761-773.

KATO, M., Y. NAKAMURA and T. TSUNODA, 2008 MOCSphaser: a haplotype inference tool from a mixture of copy number variation and single nucleotide polymorphism data. Bioinformatics **24:** 1645-1646.

KOMURA, D., F. SHEN, S. ISHIKAWA, K. R. FITCH, W. CHEN *et al.*, 2006 Genome-wide detection of human copy number variations using high-density DNA oligonucleotide arrays. Genome Res. **16:** 1575-1584.

KORN, J. M., F. G. KURUVILLA, S. A. MCCARROLL, A. WYSOKER, J. NEMESH *et al.*, 2008 Integrated genotype calling and association analysis of SNPs, common copy number polymorphisms and rare CNVs. Nat. Genet. **40:** 1253-1260.

MCCARTHY, S. E., V. MAKAROV, G. KIROV, A. M. ADDINGTON, J. MCCLELLAN *et al.*, 2009 Microduplications of 16p11.2 are associated with schizophrenia. Nat. Genet. **41:** 1223-1227.

PIQUE-REGI, R., J. MONSO-VARONA, A. ORTEGA, R. C. SEEGER, T. J. TRICHE *et al.*, 2008 Sparse representation and Bayesian detection of genome copy number alterations from microarray data. Bioinformatics **24:** 309-318.

SACHSE, C., J. BROCKMOLLER, S. BAUER and I. ROOTS, 1997 Cytochrome P450 2D6 variants in a Caucasian population: allele frequencies and phenotypic consequences. Am. J. Hum. Genet. **60:** 284-295.
