# Supplementary material for: Inferring Haplotypes of Copy Number Variations From High-Throughput Data With Uncertainty
Source: G3 (Bethesda). 2011 Jun 1;1(1):35–42. doi: 10.1534/g3.111.000174 (PMC3276117; doi:10.1534/g3.111.000174)
Supplement: Supporting Information [file supp_1.1.35_TableS1.pdf]

Table S1 Functional comparison with previous phasing tools

	Variation type[a]			Representation type[b]	
	ICN	SNVC	SNP	Logical representation	Likelihood representation
MOCSphaser	**Yes**	No	**Yes**	**Yes**	No
CNVphaser	Only 1 site	**Yes**	No	No	No
CNVphaserPro	**Yes**	**Yes**	**Yes**	**Yes**	**Yes**

[a] "Yes" means that a tool can handle not only one site but also multiple sites.

[b] "Logical representation" means that ICNs can be expressed as or-type and greater-type copy numbers (KATO *et al.* 2008) such as "2 OR 3 copies" and ">4 copies." The most characteristic point of CNVphaserPro is the ability to handle likelihood representation.
