# Supplementary material for: Inferring Haplotypes of Copy Number Variations From High-Throughput Data With Uncertainty
Source: G3 (Bethesda). 2011 Jun 1;1(1):35–42. doi: 10.1534/g3.111.000174 (PMC3276117; doi:10.1534/g3.111.000174)
Supplement: Supporting Information [file supp_1.1.35_FileS2.pdf]

File S2

Details on generating simulation data

We made simulated datasets from known real haplotypes of Sachse et al. (Sachse *et al.* 1997), who closely performed a series of target-specific experiments (long template PCR, nested PCR-RFLP method, RFLP-Southern blotting, and allele-specific PCR) to obtain diplotypes at 14 SNVC sites in the *CYP2D6* gene for 588 individuals of a Caucasian population. To our knowledge, this is the only known haplotype set that is composed of a large number of well-characterized haplotypes.

To make an input simulated dataset, we first counted up the number of each allele over a known diplotype at each of the 14 sites for each of the 588 individuals and made an unphased genotype (*e.g.*, diplotype [A, B/A] resulted in unphased genotype AAB). Next, for each of the unphased genotypes, we used a 2-dimensional normal distribution as an error model to randomly generate signal intensities. The mean and variance of a 2-dimensional normal distribution were obtained from real experimental data measured in the Affymetrix SNP 6.0 array that were downloaded from the Birdsuite website (Korn *et al.* 2008). Birdsuite regards microarray signal intensities of two alleles (represented as "A" and "B") as outcomes from the 2-dimensional normal distributions for different unphased genotypes and outputs the means and variances of such normal distributions on the basis of the Gaussian mixture model. From the downloaded data, we randomly picked up 14 sites each of which had the calculated means and variances of multiple normal distributions that corresponded to "-" (deletion), A, B, AA, AB, BB, AAA, AAB, ABB, BBB, AAAA, AAAB, ..., and BBBB, and we assigned each of these 14 sites to each of the known 14 SNVC sites (because there were no CNV sites overlapped with the *CYP2D6* gene in the downloaded data). Then, we used an unphased genotype at an SNVC site and also the normal distribution for the same genotype to randomly generate signal intensities (*e.g.*, we used both unphased genotype AAB and 2-dimenstional normal distribution for AAB to generate random signal intensities of 447.2 for "A" and 222.7 for "B"). We used the "mvtnorm" library in R for this calculation.

Finally, we calculated the probability densities of signal intensities using the normal distributions for all possible unphased genotypes, and used them as likelihood values (*e.g.*, a likelihood value for which signal intensities 447.2 and 222.7 for "A" and "B" came from AAB was 5.5×10^{-7}, that from AAA was 1.8×10^{-5}, and *etc.*). Thus, we made an input dataset in which a likelihood value was assigned to each unphased genotype at each site for each individual.

We also made ICN datasets from the SNVC datasets. As the answer dataset, we just counted the number of copies at each of the 14 sites to make diplotypes represented in ICN for each of the 588 individuals (*e.g.*, diplotype [A, B/A] resulted in ICN diplotype [2 copies/1 copy]). As an input dataset, we summed up allelic copy numbers to obtain total copy numbers (*e.g.*, diplotype [2 copies/1 copy] resulted in total copy number 3). To obtain likelihood values for total copy numbers, we summed up the likelihood values calculated for unphased genotypes across all unphased genotypes that had the same copy number (*e.g.*, for total copy number 3, we summed up likelihood values across AAA, AAB, ABB, and BBB since these have three copies) because events to observe such unphased genotypes are mutually exclusive. Thus, we obtained a likelihood value assigned to each total copy number at each site for each individual.
