# Supplementary material for: Inferring Haplotypes of Copy Number Variations From High-Throughput Data With Uncertainty
Source: G3 (Bethesda). 2011 Jun 1;1(1):35–42. doi: 10.1534/g3.111.000174 (PMC3276117; doi:10.1534/g3.111.000174)
Supplement: Supporting Information [file supp_1.1.35_FileS3.pdf]

File S3

Details on processing real data

For real data application, we used data collected using NimbleGen HD2 comparative genomic hybridization (CGH) array platform in the HapMap Phase3 CEU population. The data processing steps of scanning and spatial-normalization were performed in NimbleGen and the data processes of normalization between samples and GC-content correction of log2 ratios were performed at Cold Spring Harbor Laboratory. The details on the data processes are described in the literature (McCarthy *et al.* 2009). Following the previous procedure (Fridlyand *et al.* 2004; Kato *et al.* 2010; Komura *et al.* 2006), we first performed CNV segmentation and then used the median of log ratio intensities over probes in a segment for total copy numbers.

Specifically, we defined CNV segments only when CNV segments identified by two different segmentation tools (Day *et al.* 2007; Pique-Regi *et al.* 2008) were overlapped, more than six probes were included in segments, all the probes in a segment were mapped to only one chromosomal position, and the segment lengths were between 1 kb and 1 Mb. Also, we excluded some individuals with too many or too few segments (by outlier analysis: more/less than 3 × |third/first quartile – median|) to minimize individuals possibly affected by hybridization errors. Then, we selected CNV regions in each of which no individuals had multiple segments, the number of chromosomal parts with the maximum population frequency of CNV segments (*i.e.*, core fragment-site (Kato *et al.* 2010)) was only one, and the core part covered more than three fourths of the region length so that we could limit to regions with simple segment patterns (in our experience, segment patterns in a region tend to become complicated when the reference individual in array CGH, not test individuals, has CNVs). We selected the autosomal chromosomes' CNV regions where the core parts had more than five probes, and took the median of log ratio intensities over those probes for each individual both with and without CNV segments.

Next, we used the Gaussian mixture model and parameter estimation by the expectation-maximization method to calculate its means and variances, employing the "mclust" library in R. Then, we calculated the probability densities of the median log-ratio intensities for the multiple Gaussian distributions corresponding to zero to four copies to obtain likelihood values. For haplotype inference, we only used unrelated individuals (*i.e.*, parents).
